# Establishment of Novel Limbus-Derived, Highly Proliferative ABCG2^+^/ABCB5^+^ Limbal Epithelial Stem Cell Cultures

**DOI:** 10.1155/2017/7678637

**Published:** 2017-11-05

**Authors:** Eung Kweon Kim, Ga-Hyun Lee, Boram Lee, Yong-Sun Maeng

**Affiliations:** ^1^Department of Ophthalmology, Corneal Dystrophy Research Institute, Yonsei University College of Medicine, Seoul, Republic of Korea; ^2^Institute of Vision Research, Severance Biomedical Science Institute, Yonsei University College of Medicine, Seoul, Republic of Korea; ^3^Department of Obstetrics and Gynecology, Institute of Women's Life Medical Science, Yonsei University College of Medicine, Seoul, Republic of Korea

## Abstract

Homeostasis and regeneration of corneal epithelia are sustained by limbal epithelial stem cells (LESCs); thus, an LESC deficiency is a major cause of blindness worldwide. Despite the generally promising results of cultivated LESC transplantation, it has been limited by variations in long-term success rates, the use of xenogeneic and undefined culture components, and a scarcity of donor tissues. In this study, we identified the culture conditions required to expand LESCs *in vitro* and established human limbus-derived highly proliferative ABCG2^+^/ABCB5^+^ double-positive LESCs. These LESCs exhibited the LESC marker profile and differentiated into corneal epithelial cells. In addition, cultured LESCs expressed high levels of the stem cell markers Sox2, Oct4, c-Myc, and Klf4, had high telomerase activity, and had stable, normal genomes. These results suggest that our novel cultivation protocol affects the phenotype and differentiation capacity of LESCs. From the limbus, which contains a heterogenous cell population, we have derived highly proliferative ABCG2^+^/ABCB5^+^ double-positive cells with the ability to differentiate into corneal epithelial cells. This study opens a new avenue for investigation of the molecular mechanism of LESC maintenance and expansion *in vitro* and may impact the treatment of corneal disease, particularly corneal blindness due to an LESC deficiency.

## 1. Introduction

A surgical strategy for restoring the corneal epithelial surface in patients that lack sufficient limbal epithelial stem cells (LESCs) is the transplantation of ex vivo expanded LESCs, which is one of the few adult human stem cell therapies currently being used [1–4]. This therapeutic approach typically involves harvesting a small limbal sample from the patient or a donor followed by cell expansion to generate an epithelial sheet on a transplantable carrier, such as an amniotic membrane [5–10], fibrin gel, or temperature-responsive polymer [11]. Although successful repopulation of the ocular surface has been described for up to 1 year after transplantation, studies have indicated that epithelial viability is not sustained for very long [12] and that donor cells do not survive more than 9 months after transplantation [13, 14]. These failures may have resulted from depletion of LESCs in culture due to improper culture conditions. Most culture methods, including explant and airlift cultures, promote the proliferation and terminal differentiation of transient amplifying cells (TACs) rather than retaining LESCs [15]. However, long-term restoration of the damaged ocular surface requires the preservation of LESCs during culture and after grafting [4, 16]. Since the pioneering work in 1975 by Rheinwald and Green [17], studies have shown that long-term survival and serial expansion of LESCs are possible if they are cocultured with fibroblast feeder cells [18]. Three types of clonogenic cells, which give rise to holoclones, meroclones, and paraclones, were identified by clonal analysis of human keratinocytes cultured on feeder layers [19]. Holoclone-forming cells have all of the hallmarks of LESCs, including the capacity to self-renew and a high potential to proliferate, whereas meroclones and paraclones are generated by different stages of TACs and have limited capacities for proliferation. This discovery was followed by the identification of holoclone-forming cells in the limbal epithelium and the development of a culture system that enriches for LESCs by growing them clonally on feeder layers before seeding them onto fibrin gels to produce epithelial sheets [20, 21]. Consistently, keratinocytes cultured by this method have been used to restore massive epidermal defects permanently and to restore the corneal surface of patients with complete LESC deficiencies [1, 22–24]. Nevertheless, the question of whether the transplanted cell sheets actually contain LESCs has not been addressed and the widespread use of this promising cultivation technique has been hampered by the lack of a standardized cultivation protocol.

In this study, we evaluated the effects of several culture variables on the growth and retention of LESCs in culture to develop an optimal cultivation protocol that promotes the expansion and maintenance of LESCs for therapeutic applications. We developed a culture method to establish human limbus-derived, highly proliferative ABCG2^+^/ABCB5^+^ double-positive LESC cultures. The LESCs that we cultured by this method were confirmed to have the LESC marker profile and exhibited the potential to differentiate into corneal epithelial cells. Moreover, these LESCs expressed high levels of stem cell markers, including Sox2, Oct4, c-Myc, and Klf4 [25, 26], displayed high telomerase activity, and had stable, normal genomes. Using the limbus, which contains a heterogenous cell population, as a cell source and our specific culture conditions, we were able to establish a novel and highly proliferative ABCG2^+^/ABCB5^+^ double-positive stem cell population with the capacity for corneal epithelial differentiation. Thus, our proposed culture system may be essential for the long-term clinical success and stable regeneration of corneal epithelia to treat corneal blindness due to an LESC deficiency.

## 2. Materials and Methods

### 2.1. Cell Culture and Establishment of ABCG2^+^/ABCB5^+^ Double-Positive LESCs

Human corneal tissues were harvested from healthy corneas that were deposited in an eye bank after penetrating or lamellar keratoplasty. Donor confidentiality was maintained in accordance with the Declaration of Helsinki, and the research protocol below was approved by the Severance Hospital IRB Committee (CR04124) of Yonsei University.

1. Within 4 hours after penetrating keratoplasty, fresh corneoscleral rim tissue was placed in a 60 mm culture dish containing Hank's Balanced Salt Solution (HBSS) and was cut into four equal segments of the limbus (limbus portion including the small cornea region). Any remaining iris and endothelial cells were rubbed off with a cotton tip.
2. Each segment was digested with 15 mg/mL of dispase II (Roche, Rotkreuz, Switzerland) in supplemented hormonal epithelial media (SHEM; CELLnTEC Advanced Cell Systems AG, Bern, Switzerland) with 100 mM sorbitol (Sigma-Aldrich, St Louis, MO) at 4°C for 18 h to separate the stroma from the rest of the tissue.
3. Under a dissecting microscope, an already loose limbal epithelial sheet was separated from the tissue by inserting and sliding a noncutting flat stainless steel spatula into the plane between the limbal epithelium and the stroma.
4. The isolated limbal epithelial cell (LECs) clusters were seeded onto a 60 mm plate coated with 5% Matrigel (BD Biosciences, Bedford, MA) and 0.05 mg/ml human fibronectin (Sigma-Aldrich, St. Louis, MO) and cultured in CnT20 medium (CELLnTEC Advanced Cell Systems AG, Bern, Switzerland).
5. After 3 days, the LECs were cultured in 10% fetal bovine serum (FBS) Dulbecco's Modified Eagle Medium (DMEM; Invitrogen, Carlsbad, CA), and the medium was changed every 2 days.
6. After 8–10 days, highly proliferative cell colonies appeared in the culture plates.
7. Highly proliferative cell colonies were washed with PBS two times and treated with 1 mL Accutase (Sigma-Aldrich, St. Louis, MO), prewarmed at 37°C, and shaken evenly 5-6 times. The Accutase was then discarded, and cells were treated again with 1 mL Accutase and shaken evenly 4-5 times. Accutase was then discarded again, and cells were incubated for 3–5 min at 37°C in an incubator. Colonies were digested separately. Single cells were seeded onto plates coated with a matrix of Matrigel and fibronectin and cultured in DMEM containing 10% FBS. The highly proliferative cells that attached to the new plate were designated limbal epithelial stem cells (LESCs).
8. After 48 hours, The LESCs were treated with Accutase and sorted by FACS analysis using ABCG2^+^ antibodies (Abcam, Cambridge, MA) and ABCB5^+^ antibodies (Thermo fisher scientific, Rockford, IL).
9. The ABCG2^+^/ABCB5^+^ double-positive cells were seeded onto a plate coated with a matrix of Matrigel and fibronectin and cultured in 10% FBS DMEM. When the plate was full of cells, the cells were treated with Accutase and seeded onto a plate coated with a matrix of Matrigel and fibronectin and cultured in 10% FBS DMEM. Then, the ABCG2^+^/ABCB5^+^ double-positive cells were cultured using mass culture methods and were named ABCG2^+^/ABCB5^+^ double-positive LESCs.

### 2.2. Real-Time Quantitative Reverse Transcriptase PCR (Real-Time qRT-PCR)

Total RNA was isolated from corneal epithelial cells differentiated from ABCG2^+^/ABCB5^+^ LESCs with TRIzol reagent (Invitrogen, Carlsbad, CA). mRNA expression of human *GAPDH*, *Δnp63α*, *ABCG2*, *CK3*, *CK19*, *Integrin α9*, *CK12*, *OCT4*, *SOX2*, *NANOG*, *c-MYC*, and *KLF4* was measured using the Power SYBR Green RNA-to-CT™ 1-Step kit (Applied Biosystems, Foster City, CA, USA) and StepOnePlus™ (Applied Biosystems) according to the manufacturer's instructions. The PCR protocol was 48°C for 30 min, 95°C for 10 min, 40 cycles of 95°C for 15 s, and 60°C for 1 min. Expression results were based on cycle threshold (Ct) values. The differences between the Ct values for the experimental genes and the reference gene *GAPDH* were calculated and graphed as ratios of experimental RNAs to the calibrated sample. The primers used for gene amplification are listed in Supporting Information Table S1 available online at https://doi.org/10.1155/2017/7678637. Three independent experiments were performed, and statistical analysis was carried out using the Newman-Keuls multiple comparison test.

### 2.3. Immunofluorescence Staining

Cells were fixed in 3.7% formaldehyde for 20 min, permeabilized with 0.1% Triton X-100 in phosphate buffered saline (PBS), and preincubated in a blocking solution of PBS containing 5% normal donkey serum and 0.05% Tween-20. Then, cells were incubated with primary antibody for 2 h at room temperature. The primary antibodies used included anti-human p63*α* antibody (Cell Signaling, Beverly, MA), anti-human ABCG2 antibody (Abcam, Cambridge, England), anti-human ABCB5 antibody (Invitrogen, Carlsbad, CA), anti-human CK19 antibody (Abcam, Cambridge, England), anti-human CK3 antibody (Abcam, Cambridge, England), and anti-human desmoglein 3 antibody (Novus Biologicals, Littleton, Co.). Then, cells were labeled with a fluorescein-conjugated secondary antibody (Molecular Probes, Eugene, OR) and nuclei were counterstained with 4′6-diamidino-2-phenylindole (DAPI). Samples were observed with a fluorescence microscope (Olympus, Tokyo, Japan).

### 2.4. Differentiation of LESCs into Corneal Epithelial Cells

ABCG2^+^/ABCB5^+^ LESCs were seeded in a 12-well plate at a density of 3 × 10^4^ cells per well and cultured in 10% FBS DMEM. At 90% cell confluence, DMEM was replaced with CnT30 medium (CELLnTEC Advanced Cell Systems AG). Negative control cultures were maintained in 10% FBS DMEM. Culture media was changed every 2 days for 5 days, and then the cells were fixed in 3.7% formaldehyde and immunostained with the appropriate antibodies.

### 2.5. Differentiation of LESCs into Corneal Epithelial Cells on Transwell Filters

ABCG2^+^/ABCB5^+^ LESCs (1 × 10^5^) were seeded onto 0.4 *μ*m 12-well transwell filters in 10% FBS DMEM. At 90% cell confluence, DMEM was replaced with CnT30 medium. Negative control cultures were maintained in 10% FBS DMEM. Cell differentiation was performed under immersed conditions. Culture media was changed every 2 days for 5 days, and then the cells were fixed in 3.7% formaldehyde and immunostained with the appropriate antibodies.

After 6 days, the transwell filters were fixed in 3.7% formaldehyde, embedded into paraffin, and sliced into 8 *μ*m-thick sections. The sections were washed in PBS, blocked in 5% normal donkey serum and 0.1% Triton X-100, and incubated overnight in primary antibody 2 h at room temperature. The primary antibodies included anti-human p63*α* (Cell Signaling), anti-human ABCG2 (Abcam), anti-human ABCB5 (Invitrogen), anti-human CK3 (Abcam), and anti-human desmoglein 3 (Novus Biologicals). Then, the sections were labeled with a fluorescein-conjugated secondary antibody (Molecular Probes), and nuclei were counterstained with DAPI. Samples were observed with a fluorescence microscope (Olympus, Tokyo, Japan).

### 2.6. Differentiation of LESCs into Corneal Epithelial Cells on Amniotic Membranes

A total of 1 × 10^5^ LESCs were seeded on a 60 mm plate and infected with GFP^+^ lentivirus (MOI = 10) for 16 h. After 24 h, the transduction efficiency was evaluated based on the number of GFP-positive LESCs scored under a fluorescence microscope. Under these conditions, the transduction efficiency was 35% ± 1.2%. However, to acquire pure GFP^+^ LESCs, LESCs were selected with puromycin (lentiviral vector containing the puromycin selection marker). Thus, we used pure GFP^+^ LESCs in this experiment. We also compared the proliferation and differentiation efficiency of LESCs infected with no lentivirus, GFP-negative lentivirus, or GFP-positive lentivirus. However, we did not find any significant differences in proliferation or differentiation of LESCs under each condition.

GFP lentivirus-infected ABCG2^+^/ABCB5^+^ LESCs (1 × 10^5^) were seeded onto 1 cm^2^ human amniotic membranes in 10% FBS DMEM. After 3 days, DMEM was replaced with CnT30 medium (CELLnTEC Advanced Cell Systems AG). Culture media was changed every 2 days for 10 days. Amniotic membranes were fixed in 3.7% formaldehyde and immunostained with the appropriate antibodies.

### 2.7. Growth Assay

For serial propagations, ABCG2^+^/ABCB5^+^ LESCs and primary LECs were seeded at densities of 4 × 10^4^ cells per well on 12-well plates coated with a matrix of Matrigel and fibronectin. After 2 days, cells were confluent and passaged to new plates at a 1 : 3 ratio, which allowed cells to achieve confluence within 2 more days. Cells were passaged every 2 days for 92 days.

### 2.8. Telomerase Activity Assay

Telomerase activity in LESCs and LECs was analyzed with the TRAPEZE® Telomerase Detection Kit (S7700-KIT; Millipore Company, Purchase, NY) according to the manufacturer's instructions. In brief, a cell pellet containing 500–1000 cells was resuspended in 50 *μ*L of 1X CHAPS lysis buffer, incubated on ice for 30 min, and centrifuged. Then, 5 *μ*L of the supernatant was transferred into a fresh tube and incubated with 5 *μ*L of master mix A, which consisted of TRAP buffer (20 mM Tris-HCl, pH 8.3, 1.5 mM MgCl_2_, 63 mM KCl, 0.05% (*v*/*v*) Tween 20, 1 mM EDTA, and 0.01% BSA; TRAPEZE Telomerase Detection Kit), dNTPs, TS primer, and dH_2_O. The reaction was incubated at 30°C for 30 min, at 94°C for 1 min, and then kept on ice. Then, 10 *μ*L of master mix C (TRAP buffer, 0.01% BSA, ACX primer, Taq polymerase, 15% glycerol, SYBR green, and dH_2_O) was added, and PCR was performed in a thermocycler (Applied Biosystems Veriti Thermal Cycler; ABI) as follows: 94°C for 2 min and 40 cycles of 94°C for 10 s, 50°C for 5 s, and 72°C for 10 s. The differences between the Ct values for the LESCs and LECs were calculated and graphed.

### 2.9. Metaphase Chromosome Spread Assay

Cells were arrested in metaphase by incubation in culture media with 0.05 *μ*g/ml colcemid (final concentration) for 1 h. The cell suspensions were then treated with 75 mM KCl for 30 min at room temperature and fixed with Carnoy's solution (3 : 1 methanol : acetic acid, *v*/*v*). To form chromosome spreads, cell suspensions were dropped onto glass slides, air-dried, and stained with DAPI. Images were obtained by fluorescence microscopy. Twenty cells were analyzed in each group.

### 2.10. Flow Cytometric Analysis of the Cell Cycle

For analysis of DNA content, 1 × 10^5^ cells were harvested, washed with PBS, resuspended in 2 ml of an ice-cold 70% ethanol and 30% PBS solution, and incubated on ice for 30 min. Cells were then harvested by centrifugation and stained with 15 *μ*g/mL propidium iodide in PBS with 0.1 mg/mL RNase A for 30 min at 37°C. At least 10,000 cells were acquired per sample. Data were collected with CellQuest™ software and analyzed with ModFitLT.

### 2.11. Clonal Analysis

Clonal expansion of ABCG2^+^/ABCB5^+^ LESCs was performed by seeding a single-cell suspension at 1 × 10^3^ cells/cm^2^ on a plate coated with a matrix of Matrigel and fibronectin and culturing cells in 10% FBS DMEM. Culture media was changed every 2 days, and colony formation was monitored daily by phase contrast microscopy. Cells were fixed in 3.7% formaldehyde and immunostained with the appropriate antibodies 8 days after seeding.

### 2.12. Statistical Analysis

All of the experiments were repeated at least three times. Data were expressed as mean ± standard error, and statistical comparisons between groups were performed by one-way ANOVA followed by the Tukey's test.

## 3. Results

### 3.1. Establishment of Highly Proliferative LESC Cultures

**Figure 1:**
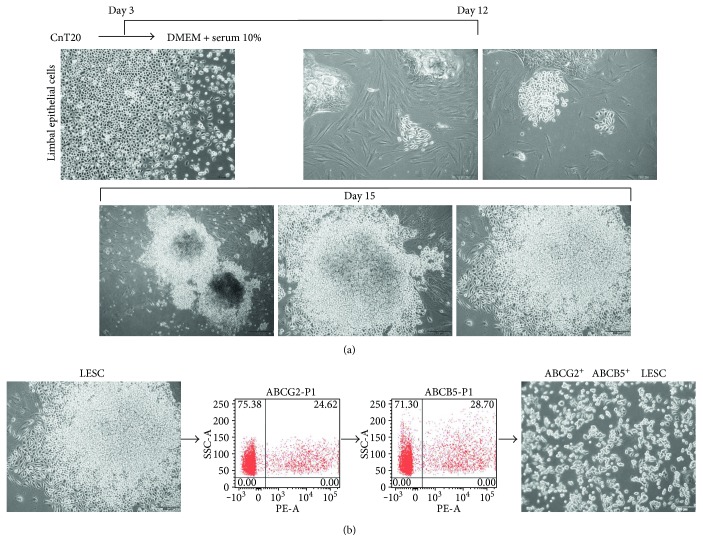
Establishment of limbus-derived highly proliferative LESCs. (a) Cultivation of highly proliferative LESCs from limbal epithelial cells. (b) Isolation of ABCG2^+^/ABCB5^+^ double-positive LESCs. Scale bars = 500 *μ*m, 200 *μ*m, and 100 *μ*m.

To obtain highly proliferative and marker-specific pure LESCs, we cultured human-derived LECs in various conditions. Matrigel has been used as an extracellular matrix for culturing LESCs [27], but Matrigel alone is not sufficient to culture LESCs. Fibronectin has also been used to culture various stem cells [28–31]. In our study, we used Matrigel, fibronectin, and a mixture of Matrigel and fibronectin as extracellular matrices for culturing LESCs. Furthermore, LESCs have been shown to maintain their stemness in cultures without serum, but in a medium with serum, LESCs differentiated into corneal epithelial cells [32]. We cultured LECs in CnT20 medium without serum and in 10% serum DMEM. A Matrigel or fibronectin matrix alone did not lead to the formation of colonies in CnT20 or in 10% serum DMEM (Supplementary Figure S1). However, highly proliferative colonies formed when a mixture of Matrigel and fibronectin was used as the matrix and when cultured for over 15 days in 10% serum DMEM (Supplementary Figure S1). To reduce the culture time and to increase the numbers of highly proliferative colonies, isolated LECs were seeded onto a plate coated with a mixture of Matrigel and fibronectin and cultured in CnT20 for 3 days. Then, the medium was replaced with 10% serum DMEM and changed every 2 days and cells were cultured for 9 days. For 9 days, medium was changed every 2 days, but cells were not passaged. During the 9 days, small colonies appeared on the plates and grew large. Among the various colony morphologies, we observed fast-growing, multilayered LESC-like colonies (Figure 1(a)).

ABCG2 and ABCB5 are known LESC markers [33, 34]. To increase the purity of the LESCs, we labeled them with antibodies to ABCG2 and ABCB5 and performed FACS analysis (Figure 1(b)). The cells isolated by FACS had the high proliferation phenotype (Figure 1(b)), and we named these cells ABCG2^+^/ABCB5^+^ double-positive LESCs. Our results demonstrate that we identified the specific culture conditions required to isolate and expand marker-specific LESCs *in vitro.*

### 3.2. Marker Analysis of LESCs and Differentiation of ABCG2^+^/ABCB5^+^ LESCs into Corneal Epithelial Cells

**Figure 2:**
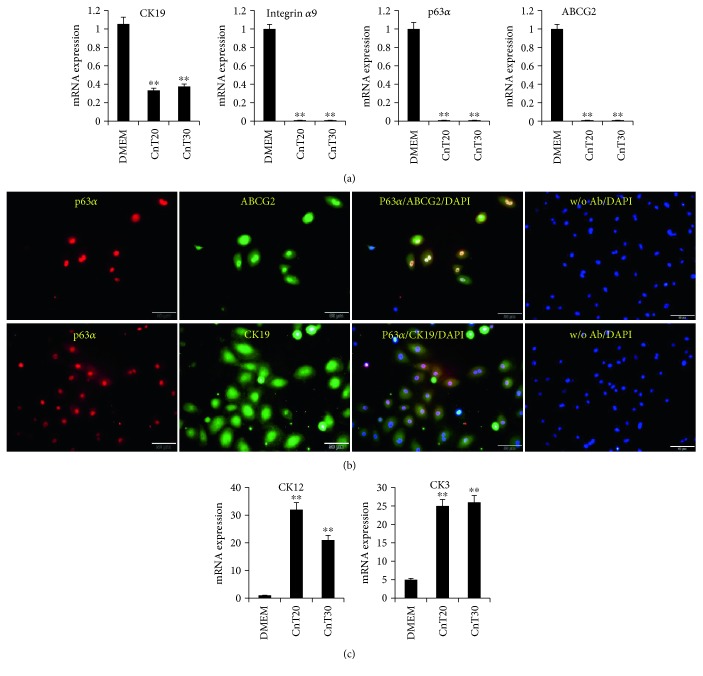
Marker analysis of ABCG2^+^/ABCB5^+^ LESCs. (a, c) Total mRNA was isolated from ABCG2^+^/ABCB5^+^ LESCs cultured in 10% DMEM, CnT20, or CnT30, and gene expression was assessed by RT-PCR. ^∗∗^*p* < 0.01 versus DMEM (b) ABCG2^+^/ABCB5^+^ LESCs were immunofluorescently stained with antibodies to p63*α*, ABCG2, and CK19. Scale bar = 50 *μ*m. w/o Ab: without primary antibody + secondary antibody.

To characterize the established ABCG2^+^/ABCB5^+^ LESCs, we analyzed their marker expression profiles by RT-qPCR and immunostaining. ABCG2^+^/ABCB5^+^ LESCs expressed LESC marker CK19, p63*α*, ABCG2, and integrin *α*9 [35–37] mRNAs when cultured in 10% serum DMEM (Figure 2(a)). Immunostaining also showed expression of p63*α*, ABCG2, and CK19 in ABCG2^+^/ABCB5^+^ LESCs (Figure 2(b)). When ABCG2^+^/ABCB5^+^ LESCs were cultured in differentiation media CnT20 and CnT30, expression of the stem cell markers CK19, p63*α*, ABCG2, and integrin *α*9 decreased (Figure 2(a)). In contrast, expression of the corneal epithelial cell markers CK3 and CK12 increased when ABCG2^+^/ABCB5^+^ LESCs were cultured in CnT20 and CnT30 (Figure 2(c)). These results demonstrate that ABCG2^+^/ABCB5^+^ LESCs express LESC-specific markers and differentiate into corneal epithelial cells.

### 3.3. Differentiation of LESCs into Corneal Epithelial Cells on Transwell Filters and Amniotic Membranes

**Figure 3:**
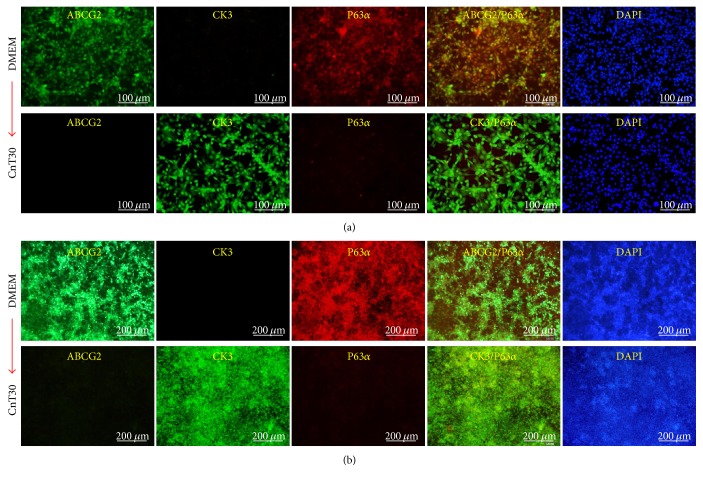
Differentiation of ABCG2^+^/ABCB5^+^ LESCs into corneal epithelial cells. (a) ABCG2^+^/ABCB5^+^ LESCs were cultured in 10% DMEM or CnT30 and stained with cell-specific markers. (b) ABCG2^+^/ABCB5^+^ LESCs were cultured on transwell filters in 10% DMEM or CnT30 and stained with cell-specific markers. Scale bars = 100 *μ*m and 200 *μ*m.

**Figure 4:**
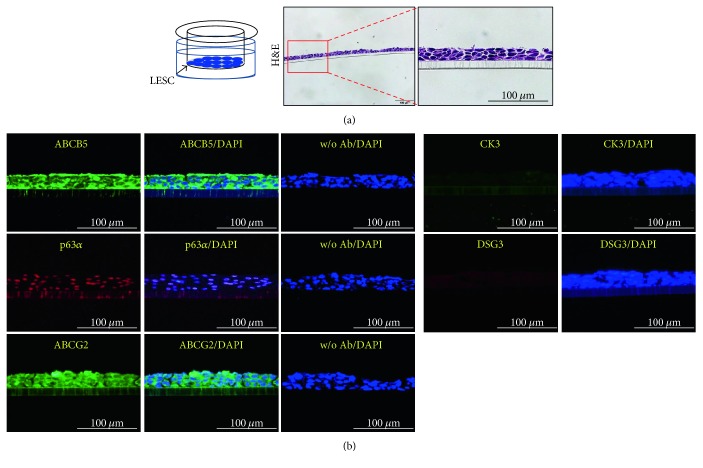
Differentiation of ABCG2^+^/ABCB5^+^ LESCs into corneal epithelial cells with a transwell system. (a) Sections of ABCG2^+^/ABCB5^+^ LESCs cultured on transwell filters were stained with hematoxylin and eosin. (b) ABCG2^+^/ABCB5^+^ LESCs were cultured onto transwell filters in 10% DMEM, and transwell sections were stained with cell-specific markers. Scale bar = 100 *μ*m. w/o Ab: without primary antibody + secondary antibody.

**Figure 5:**
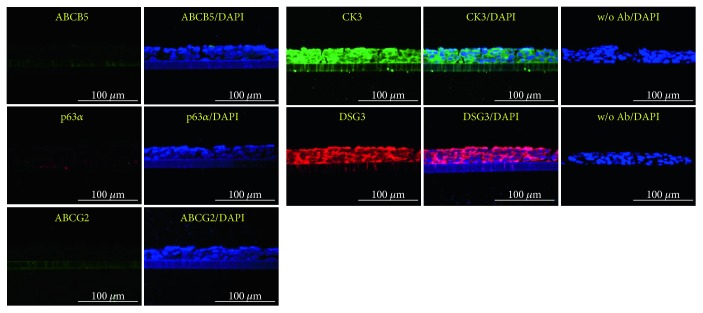
Differentiation of ABCG2^+^/ABCB5^+^ LESCs into corneal epithelial cells with a transwell system. ABCG2^+^/ABCB5^+^ LESCs were cultured onto transwell filters in CnT30, and transwell sections were stained with cell-specific markers. Scale bar = 100 *μ*m. w/o Ab: without primary antibody + secondary antibody.

**Figure 6:**
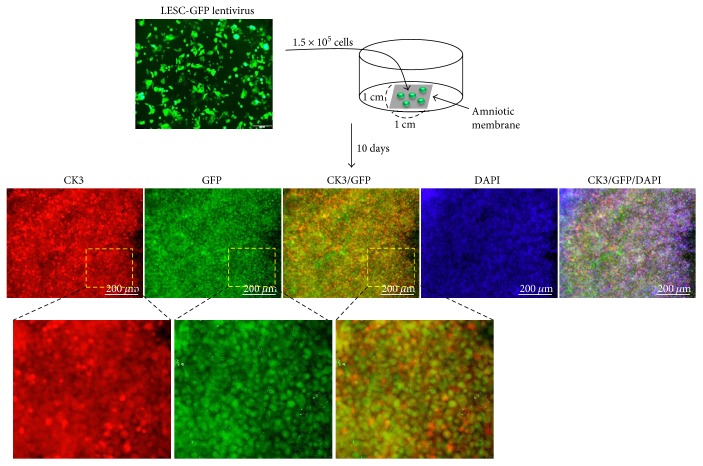
Differentiation of ABCG2^+^/ABCB5^+^ LESCs into corneal epithelial cells on an amniotic membrane. GFP-positive ABCG2^+^/ABCB5^+^ LESCs were cultured on an amniotic membrane and were stained with antibodies to CK3. Scale bar = 200 *μ*m.

LESCs have the potential to differentiate into corneal epithelial cells *in vitro* and *in vivo* [37–39]. To confirm the potential of ABCG2^+^/ABCB5^+^ LESCs to differentiate into corneal epithelial cells, we seeded ABCG2^+^/ABCB5^+^ LESCs in 12-well plates and changed the DMEM medium to CnT30 medium. When cultured in 10% serum DMEM, ABCG2^+^/ABCB5^+^ LESCs expressed the stem cell-specific markers ABCG2 and P63*α*, but when cultured in CnT30, P63*α* expression decreased and expression of the corneal epithelial cell marker CK3 increased (Figure 3(a)). Furthermore, we developed a new differentiation system that mimics *in vivo* differentiation conditions. The cornea consists of five layered cell in our body, and limbal LESCs move to the cornea and differentiate into corneal epithelial cells [39, 40]. To mimic *in vivo* conditions, we seeded ABCG2^+^/ABCB5^+^ LESCs onto 12-well transwell filters and cultured them in 10% serum DMEM or CnT30 for 5 days. For 5 days, medium was changed every 2 days, but cells were not passaged. Under these conditions, cells displayed multilayer growth without cell death. ABCG2^+^/ABCB5^+^ LESCs cultured on transwell filters in 10% serum DMEM displayed multilayer cell growth and expressed the stem cell markers ABCG2 and P63*α*. However, expression of stem cell markers decreased and expression of the corneal epithelial cell marker CK3 increased ABCG2^+^/ABCB5^+^ LESCs cultured on transwell filters in CnT30 (Figure 3(b)). To analyze the transwell-cultured ABCG2^+^/ABCB5^+^ LESCs in detail, transwell-cultured ABCG2^+^/ABCB5^+^ LESCs were embedded in paraffin and were stained immunohistochemically. Immunohistochemistry showed that the multilayered cell growth of ABCG2^+^/ABCB5^+^ LESCs on transwell filters resembles the cornea *in vivo* (Figure 4(a)). In addition, immunostaining showed that ABCG2^+^/ABCB5^+^ LESCs on transwell filters expressed the stem cell markers ABCG2, ABCB5, and P63*α* when cultured in 10% serum DMEM and expressed the corneal epithelial cell markers CK3 and desmoglein 3 and when cultured in CnT30 (Figures 4(b) and 5). Finally, we evaluated the differentiation potential of ABCG2^+^/ABCB5^+^ LESCs on amniotic membranes. GFP lentivirus-infected LESCs were seeded onto amniotic membranes and cultured for 10 days in CnT30, and they differentiated into corneal epithelial cells and expressed the corneal epithelial cell marker CK3 (Figure 6). Collectively, our results suggest that ABCG2^+^/ABCB5^+^ LESCs can differentiate into corneal epithelial cells and that our newly developed differentiation system can mimic *in vivo* differentiation and can be used to analyze the differentiation potential of LESCs.

### 3.4. Stem Cell Potential of LESCs

**Figure 7:**
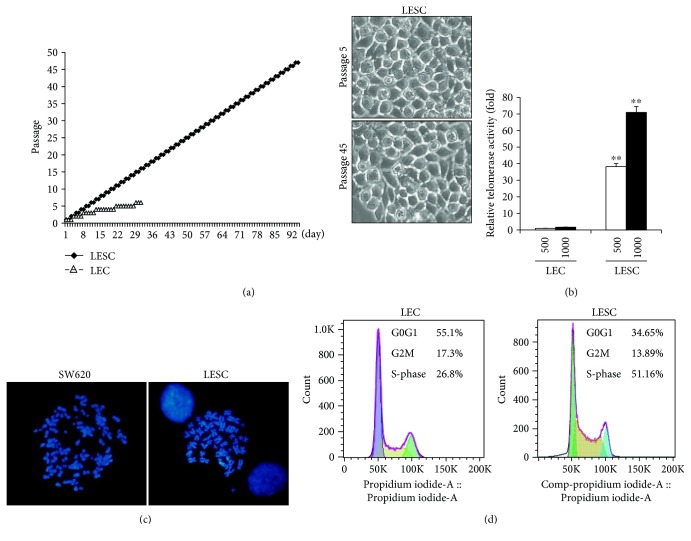
ABCG2^+^/ABCB5^+^ LESCs are potent stem cells. (a) Growth analysis of LESCs and LECs. (b) Telomerase activity in ABCG2^+^/ABCB5^+^ LESCs and in LECs (500, 1000 cells) was measured by TRAP assay. ^∗∗^*p* < 0.01 versus LEC (c) metaphase chromosome spreads of SW620 colon cancer cells and ABCG2^+^/ABCB5^+^ LESCs. (d) Cell cycle analysis of ABCG2^+^/ABCB5^+^ LESCs and LECs by propidium iodide staining and flow cytometry.

To evaluate the stem cell potential of the ABCG2^+^/ABCB5^+^ LESCs, we analyzed the growth of LECs and ABCG2^+^/ABCB5^+^ LESCs. LECs were cultured for approximately 15 days and passaged 3 times. After this time, LEC proliferation decreased and growth stopped after 30 days and 5 passages (Figure 7(a)). In contrast, ABCG2^+^/ABCB5^+^ LESCs showed continuous growth over 90 days and 50 passages (Figure 7(a)). Phase contrast image shows the maintenance of LESCs (Figure 7(a)). Furthermore, because telomerase activity is associated with cell proliferation in cultured cells [41] and in some stem cells [42], we examined telomerase activity in ABCG2^+^/ABCB5^+^ LESCs and found high telomerase activity (Figure 7(b)). However, telomerase activity was not detected in primary LECs (Figure 7(b)). After culturing ABCG2^+^/ABCB5^+^ LESCs for 90 days, metaphase chromosome spread analysis was performed to detect chromosome instabilities. The chromosome spread assay has the powerful ability to analyze individual cells for genome aberrations, including insertions, deletions, and rearrangements involving one or more chromosomes [43]. No genomic insertions, deletion, or rearrangements were detected in ABCG2^+^/ABCB5^+^ LESCs; however, genetic abnormalities were detected in SW620 colon cancer cells (Figure 7(c)). In addition, cell cycle analysis by flow cytometry showed an increase in the S-phase of ABCG2^+^/ABCB5^+^ LESCs, which has also been seen in some stem cells, but the cell cycle of LECs was found to be normal (Figure 7(d)).

Some stem cells express core transcription factors, such as Oct4, Sox2, Nanog, c-Myc, and Klf4 [44–46]. To assess expression of core transcription factors in ABCG2^+^/ABCB5^+^ LESCs, we isolated mRNA from ABCG2^+^/ABCB5^+^ LESCs cultured in different conditions and performed RT-qPCR. ABCG2^+^/ABCB5^+^ LESCs expressed Oct4, Sox2, c-Myc, and Klf4 mRNAs when cultured in 10% serum DMEM, but expression of these markers decreased when the cells were cultured in CnT20 or CnT30 differentiation media (Supplementary Figure S2). To further elucidate the stem cell character of ABCG2^+^/ABCB5^+^ LESCs, we examined the colony-forming activity of ABCG2^+^/ABCB5^+^ LESCs. Immunostaining showed strong colony formation by ABCG2^+^/ABCB5^+^ LESCs (Supplementary Figure S3) indicating that ABCG2^+^/ABCB5^+^ LESCs have significant stem cell activity and may be used to regenerate corneal epithelia. Moreover, ABCG2^+^/ABCB5^+^ LESCs may be multipotent and may be able to differentiate into other cell lineages in addition to corneal epithelial cells.

## 4. Discussion

Many researchers have attempted to retain LESCs in culture, but have been unsuccessful. Since the pioneering work in 1975 by Rheinwald and Green [17], studies have shown that long-term survival and serial expansion of LESCs are possible if they are cocultured with fibroblast feeder cells. Nevertheless, the question of whether the transplanted cell sheets actually contain LESCs has not been addressed and the widespread use of this promising cultivation technique has been hampered by the lack of a standardized cultivation protocol. To expand cells and generate epithelial sheets, fibrin gels, temperature-responsive polymers, and amniotic membranes have been used [11], but epithelial cell viability was not sustained for very long [12], and no donor cells survived 9 months after transplantation [13, 14]. These failures may have resulted from depletion of LESCs in culture due to improper culture conditions. Rather than favoring retention of LESCs, most culture methods promote the proliferation and terminal differentiation of transient amplifying cells (TACs) [15]. Long-term restoration of damaged ocular surfaces requires retention of a sufficient amount of LESCs during culturing and after grafting [4, 16] to ensuring successful regeneration of the ocular surface [47, 48].

In this study, we developed an optimal method to expand and increase the survival and proliferation of LESCs derived from a small limbal biopsy. Matrigel and fibronectin were used as matrices for culturing LESCs and other stem cells [27–31], but Matrigel or fibronectin alone is not sufficient to culture LESCs. In this study, highly proliferative LESC colonies formed when we used a mixture of Matrigel and fibronectin as the extracellular matrix.

LESCs are located in the basal region of the limbus and require a specific environment for survival and growth. To mimic the in vivo environment, we used various extracellular matrix components (Matrigel, collagen, gelatin, fibronectin, fibrin, etc.) for the in vitro culture of LESCs. However, we did not identify the optimal conditions for LESC culture. Matrigel has been used as an extracellular matrix for culturing LESCs [27]. However, Matrigel alone is not sufficient for the culture of LESCs under our conditions. We have had much experience in the culture of many types of stem cells (mesenchymal stem cells, endothelial stem cells, neuronal stem cells, embryonic stem cells, etc.) and have found that fibronectin has good effects on stem cell proliferation. Therefore, we mixed Matrigel and fibronectin, and highly proliferative LESC colonies formed when we used this mixture as the extracellular matrix. Other combinations of extracellular matrix components did not yield sufficient LESC culture. These results suggested that stimulation by fibronectin may recover the signal required for LESC growth, which could not be obtained by Matrigel alone.

In addition, LESCs are known to maintain their stemness in a medium without serum, but a medium with serum induces differentiation of LESCs into corneal epithelial cells [32]. We cultured LECs in CnT20 medium without serum and in 10% serum DMEM without extracellular matrices and did not observe highly proliferative cell phenotypes. However, highly proliferative cell colonies formed when LECs were cultured with a matrix of Matrigel and fibronectin in 10% serum DMEM indicating that specific culture conditions, including the compositions of the extracellular matrix and the cell culture medium, are required for efficient growth of undifferentiated LESCs. In this study, we demonstrated that corneal LESCs can be consistently expanded *in vitro* using a mixed extracellular matrix and a medium containing serum. Meyer-Blazejewska et al. proposed an improved culture protocol in 2010 [37]. There are three major differences between our method and the method of Meyer-Blazejewska et al. First, we cultured the cells on a mixture of Matrigel and fibronectin, whereas they cultured the cells on a 3T3 feeder cell layer. Second, we used CnT20 and DMEM containing 10% serum, whereas they used MCDM151, Epilife, DMEM/F12, PCT, or D-KSFM with several growth factors. Third, we used transwell filters and amniotic membranes for in vitro differentiation, whereas they used fibrin gel. Most importantly, the clonal growth phenotype was very different. Under our conditions, LESCs showed rapid growth and multilayered colonies on the plates. However, they showed only monolayer colonies. Collectively, these results suggested that our method for cultivation of ABCG2^+^/ABCB5^+^ LESCs was different from that of Meyer-Blazejewska et al. and that our established LESCs exhibited different characteristics, although some markers were similar.

The isolated and cultured ABCG2^+^/ABCB5^+^ LESCs retained their viability and stemness as confirmed by the presence of the stem cell markers CK19, p63*α*, ABCG2, and integrin *α*9. We confirmed *in vitro* differentiation of LESCs into corneal epithelial cells by the presence of the markers CK12, CK3, and desmoglein3. These results suggest that ABCG2^+^/ABCB5^+^ LESCs express LESC-specific markers and can differentiate into corneal epithelial cells. Moreover, our newly developed transwell filter differentiation system mimics *in vivo* differentiation and may be used to analyze the differentiation potential of LESCs *in vivo.*

The ABCG2^+^/ABCB5^+^ LESCs that we established displayed powerful stem cell activity, continuous growth, and high telomerase activity without chromosome instability. In addition, cell cycle analysis by flow cytometry showed that the S-phase of ABCG2^+^/ABCB5^+^ LESCs increased, which has been observed in other stem cells, but the cell cycle of LECs remained normal. In general, fate-determined normal cells (limbal epithelial cells, fibroblasts, endothelial cells, skin epithelial cells, etc.) exhibited a slow cell cycle, and about 20–30% of cells were in the S-phase, similar to LECs. However, stem cells (mesenchymal stem cells, embryonic stem cells, etc.) showed a rapid cell cycle and increased percentage of cells in the S-phase (over 50% of cells were in the S-phase). ABCG2^+^/ABCB5^+^ LESCs grew very rapidly and showed an increase in the proportion of cells in the S-phase (over 50% of cells were in the S-phase), similar to other stem cells. Therefore, we suggested that ABCG2^+^/ABCB5^+^ LESCs had stem cell characteristics.

Moreover, ABCG2^+^/ABCB5^+^ LESCs expressed the core transcription factors Oct4, Sox2, c-Myc, and Klf4, which are also expressed in multipotent stem cells. These data indicate that the ABCG2^+^/ABCB5^+^ LESCs that we established have powerful stem cell activity and may be used to regenerate corneal epithelia. Moreover, ABCG2^+^/ABCB5^+^ LESCs may be multipotent and may be able to differentiate into additional cell lineages.

In conclusion, our results show that with the appropriate methods, including the appropriate matrix and medium, human limbus-derived, highly proliferative ABCG2^+^/ABCB5^+^ double-positive LESCs can be cultured. The cultured LESCs exhibited the LESC marker profile and the ability to differentiate into corneal epithelial cells. Moreover, the LESCs expressed high levels of the multipotent stem cell markers Sox2, Oct4, c-Myc, and Klf4, displayed high telomerase activity, and were found to have a stable, normal genome. These results suggest that our novel culture system may be essential for long-term clinical success and stable regeneration of corneal epithelia to treat corneal blindness due to an LESC deficiency.

## 5. Conclusions

In summary, we described an improved cultivation protocol using biopsies from the limbus, appropriate extracellular matrices, and appropriate culture media to clonally expand marker-specific-isolated stem cells and to subsequently subcultivate highly proliferative cell colonies on a mixed Matrigel and fibronectic extracellular matrix in a defined environment to support the expansion and retention of stem cells. Whether this culture technique enhances the therapeutic potential of LESC transplantation remains to be evaluated. Nevertheless, this culture system may represent a new starting point for establishing a true stem cell-based therapy for long-term ocular surface reconstruction. Moreover, for extended survival of stem cells in a cultured graft, factors that reproduce the niche environment must to be integrated into the culture system in the future.

## Supplementary Material

Figure S1: Cultivation of LECs in various culture conditions. Isolated LECs cultured on 5% matrigel, 0.05 mg/ml fibronectin, or a mixture of 5% matrigel + 0.05 mg/ml fibronectin in CnT20 or 10% DMEM for 15 days. Scale bar = 100 μm. Figure S2: Stem cell marker analysis of ABCG2+/ABCB5+ LESCs. Total mRNA was isolated from ABCG2+/ABCB5+ LESCs cultured in 10% DMEM, CnT20, or CnT30 and gene expression was assessed by RT-PCR. ∗∗ p < 0.01 vs DMEM. Figure S3: Colony formation analysis of ABCG2+/ABCB5+ LESCs. ABCG2+/ABCB5+ LESCs were seeded and cultured for 8 days. Colony formation was monitored by staining with antibodies to ABCG2 and P63α. Scale bar = 500 μm and 200 μm. Supplementary Table S1.
