# Supplementary material for: Establishment of Novel Limbus-Derived, Highly Proliferative ABCG2+/ABCB5+ Limbal Epithelial Stem Cell Cultures
Source: Stem Cells Int. 2017 Nov 5;2017:7678637. doi: 10.1155/2017/7678637 (PMC5694571; doi:10.1155/2017/7678637)

Supplementary Figures

Figure S1: Cultivation of LECs in various culture conditions. Isolated LECs cultured on 5% matrigel, 0.05 mg/ml fibronectin, or a mixture of 5% matrigel + 0.05 mg/ml fibronectin in CnT20 or 10% DMEM for 15 days. Scale bar = 100 μm.

Figure S2: Stem cell marker analysis of ABCG2+/ABCB5+ LESCs. Total mRNA was isolated from ABCG2+/ABCB5+ LESCs cultured in 10% DMEM, CnT20, or CnT30 and gene expression was assessed by RT-PCR. ** $p < 0.01$ vs DMEM.

Figure S3: Colony formation analysis of ABCG2+/ABCB5+ LESCs. ABCG2+/ABCB5+ LESCs were seeded and cultured for 8 days. Colony formation was monitored by staining with antibodies to ABCG2 and P63α. Scale bar = 500 μm and 200 μm.

Supplementary Table S1

p63α	(sense)- accatcagaagatggtgcga	OCT4	(sense)- aagcgatcaagcagcgacta
	(antisense)- ccaggttcgtgtactgtggc		(antisense)- gaagtgagggctcccatagc
ABCG2	(sense)- ttggctgtcatggcttcagt	SOX2	(sense)- gaccagctcgcagacctaca
	(antisense)- ctgttgcattgagtcctggg		(antisense)- gaagaggtaaccacaggggg
CK3	(sense)- gggcgaggagtacaggatgt	NANOG	(sense)- cagaaaaacaactggccgaa
	(antisense)- gccgtaacctcctccatagc		(antisense)- ggtctggttgctccacattg
CK19	(sense)- acacactggcagaaacggag	C-MYC	(sense)- agcaacaaccgaaaatgcac
	(antisense)- gacttgatgtccatgagccg		(antisense)- ccgttttagctcgttcctcc
Integrin α9	(sense)- atgggaacccagaagaggtg	KLF4	(sense)- taccaagagctcatgccacc
	(antisense)-cagcagcaggaagatgagga		(antisense)- ctttgtgtaggttttgccgc
CK12	(sense)- ggagattgagacctaccgcc	GAPDH	(sense)- atggggaaggtgaaggtcg
	(antisense)- atttttcgggttttggttgg		(antisense)- ggggtcattgatggcaacaata

Supplementary Figure 1

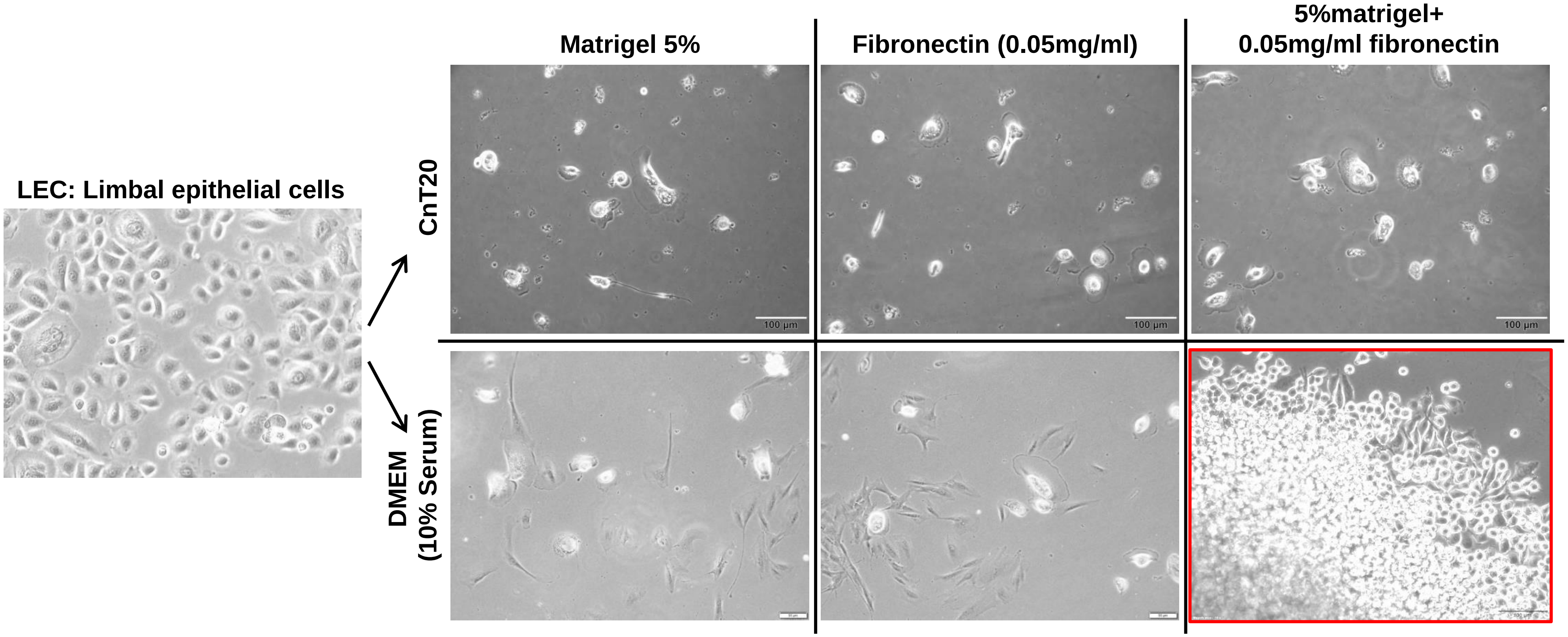

Supplementary Figure 2

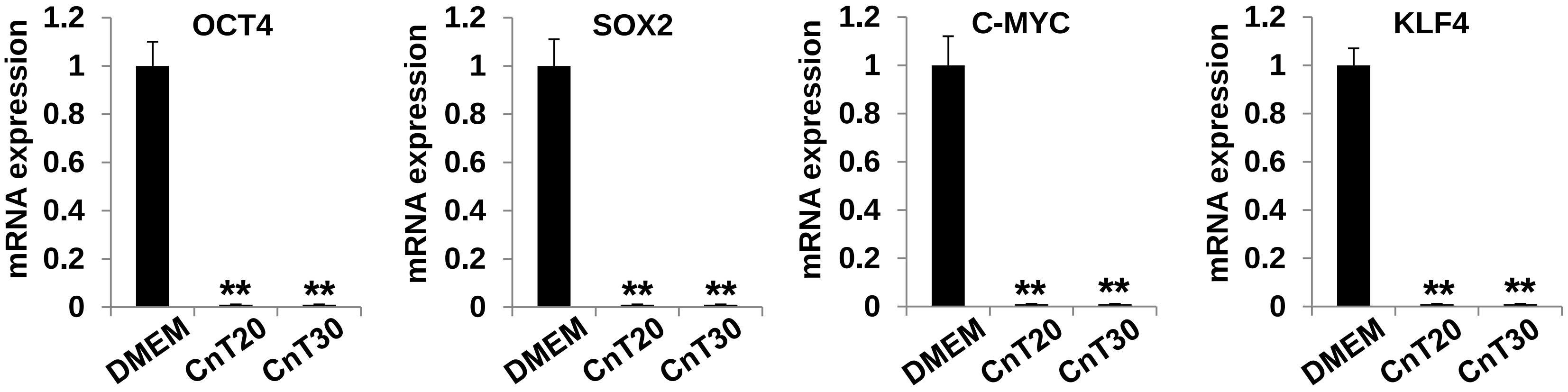

Supplementary Figure 3

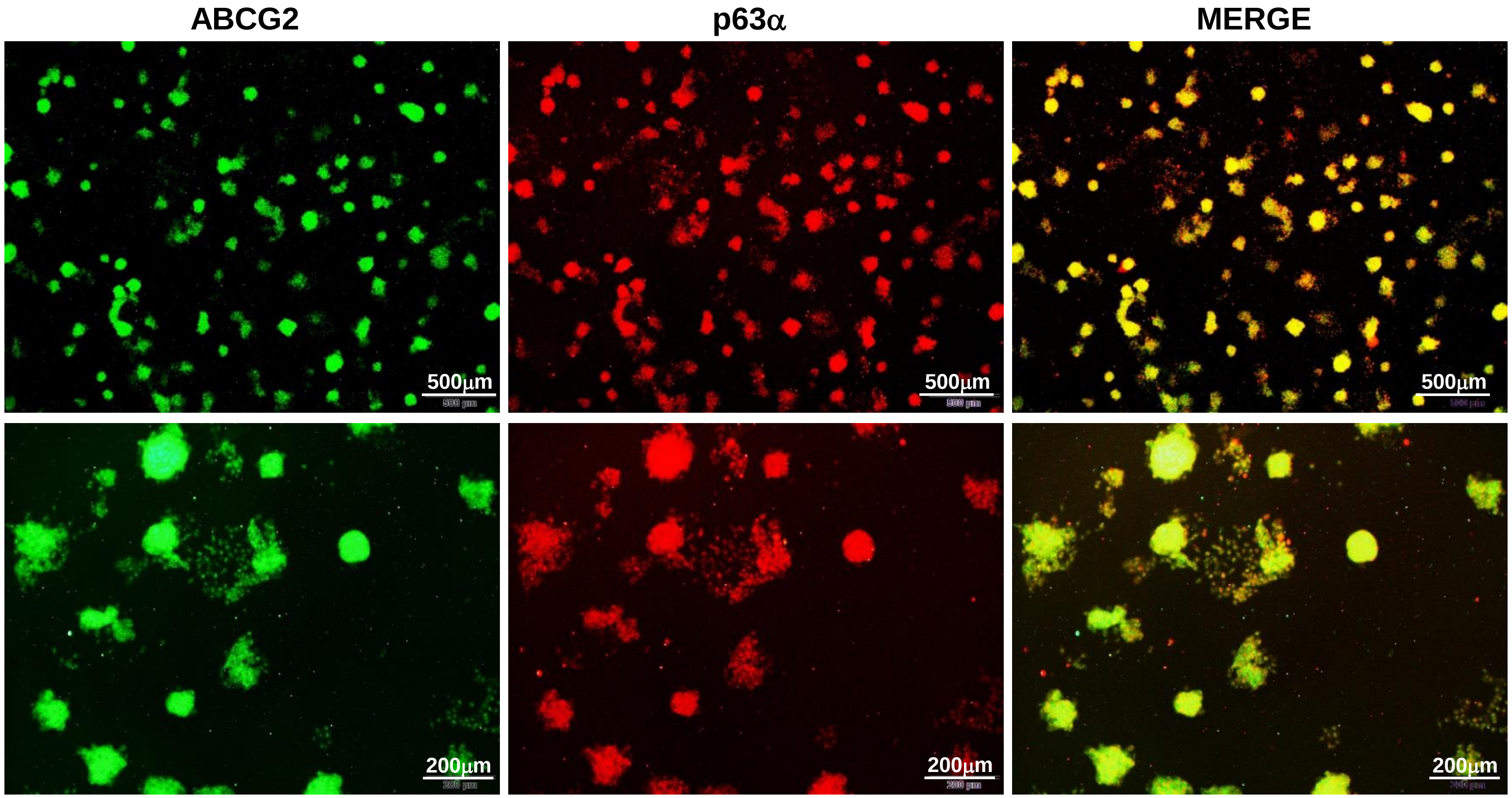

Supplement: Supplementary file 1 — Figure S1: Cultivation of LECs in various culture conditions. Isolated LECs cultured on 5% matrigel, 0.05 mg/ml fibronectin, or a mixture of 5% matrigel + 0.05 mg/ml fibronectin in CnT20 or 10% DMEM for 15 days. Scale bar = 100 μm. Figure S2: Stem cell marker analysis of ABCG2+/ABCB5+ LESCs. Total mRNA was isolated from ABCG2+/ABCB5+ LESCs cultured in 10% DMEM, CnT20, or CnT30 and gene expression was assessed by RT-PCR. ∗∗ p < 0.01 vs DMEM. Figure S3: Colony formation analysis of ABCG2+/ABCB5+ LESCs. ABCG2+/ABCB5+ LESCs were seeded and cultured for 8 days. Colony formation was monitored by staining with antibodies to ABCG2 and P63α. Scale bar = 500 μm and 200 μm. Supplementary Table S1. [file 7678637.f1.pdf]
